# Elevation Mechanisms and Diagnostic Consideration of Cardiac Troponins under Conditions Not Associated with Myocardial Infarction. Part 2

**DOI:** 10.3390/life11111175

**Published:** 2021-11-03

**Authors:** Aleksey M. Chaulin

**Affiliations:** 1 Department of Cardiology and Cardiovascular Surgery, Samara State Medical University, 443099 Samara, Russia; a.m.chaulin@samsmu.ru; Tel.: +7-(927)-770-25-87; 2 Department of Histology and Embryology, Samara State Medical University, 443099 Samara, Russia

**Keywords:** cardiac troponins, diabetes mellitus, arterial hypertension, hereditary cardiomyopathies, atrial fibrillation, supraventricular tachycardia, acute aortic dissection, strokes, subarachnoidal hemorrhage, false positive

## Abstract

This article proceeds with a discussion of the causes and mechanisms of an elevation in cardiac troponins in pathological conditions not associated with acute myocardial infarction. The second part of the article discusses the causes and mechanisms of cardiac troponins elevation in diabetes mellitus, arterial hypertension, hereditary cardiomyopathies, cardiac arrhythmias (atrial fibrillation, supraventricular tachycardia), acute aortic dissection, and diseases of the central nervous system (strokes, subarachnoidal hemorrhage). The final chapter of this article discusses in detail the false-positive causes and mechanisms of elevated cardiac troponins.

## 1. Causes and Mechanisms of Elevated Cardiac Troponins in Diabetes Mellitus

Modern high-sensitive methods for the determination of cardiac troponins can reveal relatively minor and reversible damage to cardiomyocytes peculiar to certain pathologies (e.g., diabetes mellitus, arterial hypertension), which increase the risk of life-threatening diseases [1,2,3,4]. Diabetes mellitus is considered one of the key risk factors for the development and progression of cardiovascular diseases. It often causes various microvascular (retinopathy, nephropathy, neuropathy) and macrovascular pathologies (ischemic heart disease (IHD), acute myocardial infarction (AMI), stroke, peripheral arterial disease) [1,5,6]. Several studies proved that elevated cardiac troponin I concentrations indicative of asymptomatic myocardial injury predict the risk of major adverse cardiovascular events (MACE) [7,8,9]. Eubanks et al. recorded elevated troponin I levels in 24 of 264 diabetes mellitus patients, with no evidence of an acute coronary syndrome (ACS). Regression analysis shows that elevated levels are independently associated with MACE in the long term. The authors also noted that almost half of the patients with elevated troponin I levels had a history of coronary artery disease. Based on these data, researchers believe that damage to cardiomyocytes is caused by ischemia resulting from an imbalance between myocardial oxygen demand and oxygen delivery [8]. So, being associated with ischemic heart disease, oxygen delivery to the myocardium through the coronary arteries decreases, and with an underlying increase in the levels of contrainsular hormones, myocardial oxygen demand increases. In another large study of 1275 intent-to-treat patients with diabetes mellitus without signs of ACS, increased levels of cardiac troponin I in serum were recorded in 22% of patients. Researchers noted a close relationship between elevated troponin I concentrations and the risk of developing MACE over the next 3 years (odds ratio (OR) = 1.98; 95% confidence interval (CI) 1.48–2.65; *p* < 0.001) [9]. Another evidence of the development of asymptomatic myocardial damage in diabetes mellitus was obtained in the ARIC (Atherosclerosis Risk in Communities) study. In this study, elevated serum levels of high-sensitive troponin T (hs-cTnT) were associated with chronic hyperglycemia [10].

A study conducted by Yiu K. and colleagues proved that asymptomatic myocardial injury detected by high-sensitive cardiac troponin I (hs-cTnI) is associated with increased arterial stiffness [11]. The formation of increased stiffness of the arterial wall in diabetes mellitus is conditioned by several mechanisms, such as increased oxidative stress, activation of endothelial cell apoptosis, endothelial dysfunction, and endothelial cells’ regenerative potential deterioration [12,13,14,15]. The high stiffness of the arterial wall causes asymptomatic myocardial injury through several mechanisms. This way, a decrease in the elasticity of the aortic wall determines systolic pressure, and preload of the left ventricle increases, which excessively loads the left ventricular myocardium during systole and conditions its hypertrophy. Left ventricular hypertrophy may be associated with increased levels of hs-cTnT [16], which may indicate that more cardiac troponin molecules are released from the hypertrophied myocardium [17]. This circumstance is also confirmed by the gender characteristics of the levels of hs-cTnT and hs-cTnI, which, according to many researchers, are due to the difference in myocardial mass (higher in men than in women) [18,19,20]. In addition to this mechanism, an increase in the stiffness of the arterial wall promotes a decrease in diastolic blood pressure and diastolic perfusion pressure in the coronary arteries, inducing a decrease in myocardial tissue perfusion and its ischemia [21,22]. It should also be noted that myocardial ischemia caused by the increased stiffness of the arterial wall develops regardless of the presence of another complication of diabetes mellitus, namely, atherosclerosis of the coronary arteries, which contributes to the formation of coronary artery disease, narrowing of the lumen of the coronary vessels, and a subsequent decrease in oxygen delivery. The comprehensive effect of these mechanisms further enhances the ischemia of the striated cardiac muscle tissue.

Another mechanism that may lead to an increase in the concentration of hs-cTnT and hs-cTnI in the blood serum in diabetes mellitus is the disturbed elimination of protein molecules of cardiac troponins from the blood serum. Kidney damage (nephropathy) is a frequent complication of diabetes mellitus and, without optimal treatment, further leads to the formation of chronic kidney disease (CKD) [23]. CKD is accompanied by a decrease in the glomerular filtration rate (GFR), which reduces the clearance of cardiac troponin molecules and promotes their accumulation in the blood serum [24,25,26].

Alongside the determination of highly sensitive troponins (hs-cTnT and hs-cTnI) in blood serum, it is possible to study them in other biological fluids; in particular, in the patients’ oral fluid [27,28,29,30] and urine [31]. Obtaining these biomaterials, in contrast to blood, is carried out in a non-invasive way; therefore, it is a more convenient diagnostic method on an outpatient basis. In a recent study, Chen and colleagues examined the predictive value of hs-cTnI in urine in patients with diabetes mellitus (n = 378). According to multivariate logistic regression analysis, urinary hs-cTnI levels > 4.1 pg/mL were associated with an increased risk of adverse cardiovascular events during the 3-month follow-up period [31]. It should be noted that urine and oral fluid troponins determination is not used in actual clinical practice so far. It is explained by the relative paucity of clinical studies carried out to date, which does not allow for a reliable judgment of the optimal diagnostic/prognostic value of cardiac troponins in urine and/or oral fluid. Nevertheless, obtaining these biological fluids has several pronounced advantages, such as non-invasiveness, painlessness, reduced risk of contracting blood-borne infections, as well as the ease of obtaining this biomaterial (no special medical skills are required, such as, for example, to obtain venous blood). According to the authors, further research in this area on larger patient samples is needed to confirm the results obtained, to possibly later introduce this method into outpatient clinical practice [27,28,29,30,31].

All the above allows distinguishing the following mechanisms for the elevation in cardiac troponins in diabetes mellitus: (1) myocardial ischemia caused by an imbalance between oxygen demand and delivery; (2) myocardial hypertrophy caused by an increase in the stiffness of the arterial wall; and (3) a decrease in the elimination of cardiac troponin molecules from the blood serum, due to a decrease in GFR in CKD. Since elevated serum and urine levels of cardiac troponins in diabetes mellitus are associated with the risk of adverse outcomes, they can be considered useful prognostic biomarkers to optimize the management of patients with diabetes mellitus.

## 2. Causes and Mechanisms of Elevated Cardiac Troponins in Arterial Hypertension

Arterial hypertension (AH) is a significant risk factor for the development of CVD, which has a tremendous impact on the quality of life and mortality of the population on our planet. Thus, according to a large study by Lawes et al., hypertension causes 47% of cases of ischemic heart disease/AMI and 54% of strokes annually [32]. The prevalence of hypertension varies depending on different regions and populations. For example, the prevalence of hypertension among residents of Canada is on average 22% [33]. Moreover, in the indigenous population of the province of Manitoba, the prevalence of hypertension reached 35% among Canadian aborigines and up to 28% among Canadian mestizos [34,35]. However, according to Caligiuri et al., the real prevalence of hypertension is about twice as high as in the official reports present. Thus, the authors surveyed 1097 healthy Canadian residents (visitors to shopping centers, various social events) and found increased blood pressure levels in half of them. At the same time, in 2% of the examined individuals, the blood pressure values exceeded 180/110 mm Hg, which required the provision of emergency care [36]. Particular concern was also caused by the fact that most of the examined persons were not aware of high blood pressure and took no antihypertensive medication [36]. In the United States, hypertension is diagnosed in about one in three adults, and it is estimated that in 10% of Americans hypertension remains underdiagnosed [37]. This makes hypertension not only widespread but also an insidious pathology, which can be asymptomatic for a long time and subsequently lead to dangerous complications in the form of acute CVD (AMI, strokes, etc.).

Several studies have noted that arterial hypertension is a significant cause of elevated cardiac troponins in patients without AMI [2,38,39,40,41,42]. The prevalence of elevated levels of cardiac troponins in hypertension patients varies according to the data of different studies and, most likely, is due to the difference in the methods of determination used (sensitivity of immunoassays) and the basic clinical characteristics of the intent-to-treat patients. Thus, Afonso et al. in their retrospective study recorded an increase in serum levels of cardiac troponins in 32.3% of patients with hypertension [41]. In two other clinical studies, elevated serum concentrations of cardiac troponins were found in 7% and 15% of patients with arterial hypertension [42,43]. Significant differences in the prevalence and elevation degree of cardiac troponins in arterial hypertension patients depend on the sensitivity of the applied laboratory methods. Elevated cardiac troponin levels are associated with a long-term risk of developing MACE. So, according to Pattanshetty et al., in patients with elevated serum troponin I levels, the risk of MACE occurrence was significantly higher than in troponin-negative patients (OR = 2.77; 95% CI: 1.79–4, 27; *p* < 0.001) [44].

Modern high-sensitive immunoassays allow the detection of asymptomatic myocardial damage at the first stages of hypertension and the identification of individuals at high risk of developing this pathology. Thus, in a recent large study, elevated serum hs-cTnT levels in healthy patients were associated with a higher risk of hypertension in the long term. In addition, elevated hs-cTnT concentrations (≥14 ng/L) in healthy patients without obvious signs of CVD were associated with the risk of developing left ventricular hypertrophy during the 6-year follow-up period (OR = 5.19, 95% CI: 1.49–18.08) [45]. Thus, hs-cTnT may be of great importance for outpatient monitoring and identification of patients at high risk of developing arterial hypertension.

According to a study by Pervan et al. [46], urinary hs-cTnI levels are of high diagnostic value in patients with arterial hypertension. However, as noted above, the number of clinical studies devoted to the diagnostic value of urine cardiac troponins is relatively small, and therefore this type of sample (biomaterial) has not yet been validated in commercially available troponin immunoassay methods. Therefore, these findings require further refinement in larger studies.

The mechanisms of the elevation in cardiac troponins in hypertension have not been finally defined and further research is required to clarify them. Some studies distinguish the following mechanisms of increasing cardiac troponins in hypertension:

(1) Activation of the processes of apoptosis of cardiomyocytes, caused by an increase in the load on the myocardium and its stretching [47,48], or an increase in the activity of the adrenergic system [48]. These mechanisms play an important role in the pathogenesis of hypertension; therefore, their contribution to the elevation in serum levels of cardiac troponins can be considered very reasonable.

(2) Myocardial hypertrophy, conditioned by exposure to high blood pressure. The evidence for this mechanism is proved by studies that revealed the association of hypertrophy and elevated serum levels of cardiac troponins [49], as well as data on the gender characteristics of the concentrations of hs-cTnT and hs-cTnI, due to gender differences in heart muscle mass.

(3) The effect of blood pressure on the processes of elimination of cardiac troponins from blood serum may play a role in the elevation of the urinary levels of cardiac troponins [46]. So, with an increase in blood pressure, the GFR is enhanced, and, probably, more troponin molecules pass through the glomerular filter.

## 3. Causes and Mechanisms of Elevated Cardiac Troponins in Hereditary Cardiomyopathies

Molecules of cardiac troponins in cardiomyopathies have long been of considerable interest to many specialists. Thus, the widespread attention of genetic and pathophysiology scientists to the protein molecules of troponins is due to the mutations in the genes encoding these proteins that disrupt the functioning of troponins and the entire myocardium contractile apparatus, which manifests itself in the form of hereditary cardiomyopathies [50,51,52,53,54]. Alongside these genetic and pathophysiological aspects, laboratory monitoring may provide important diagnostic/prognostic value regarding the serum levels of cardiac troponins in patients with hereditary cardiomyopathies. As a rule, hereditary cardiomyopathies are accompanied by pronounced and steadily progressive disorders of the myocardium contractile function, which indicates damage and death of cardiomyocytes and the release of cytoplasmic molecules (cardiomarkers) into the blood serum. Thus, elevated levels of cardiomarkers in the blood are valuable indicators of myocardial function and further the prognosis of patients.

Japanese researchers, guided by Hamada, observed 77 patients with hypertrophic cardiomyopathy (HCM) for 20 years. The concentrations of creatine kinase-MB, lactate dehydrogenase-1, troponin T, and myosin light chains were measured in all patients. An increase in the troponin T levels was noted in only three patients and creatine kinase-MB levels in 64% of patients. The concentrations of lactate dehydrogenase-1 and myosin light chains were not significantly elevated compared with the control group. During the observation period, it was noted that in patients with increased concentrations of creatine kinase-MB, the left ventricular end-diastolic dimension (*p* < 0.0001) increased, the shortening fraction (*p* < 0.0004) decreased, and the left atrial size dimension was enlarged (*p* < 0.0001). Heart failure developed in 18 patients with elevated levels of creatine kinase-MB and only in four patients with normal concentrations of creatine kinase-MB. In addition, among patients with elevated levels of creatine kinase-MB, mortality from heart failure was significantly higher among patients with elevated levels of creatine kinase-MB [55]. Thus, this cardiomarker is of a high predictive value. The authors believe that persistently elevated serum levels of cardiomarkers indicate ongoing alteration of the myocardium, which leads to the death of patients with HCM from heart failure. When analyzing this study [55], it is also worth noting that the prevalence of elevated cardiac troponin T levels among patients with HCM was extremely low and this biomarker had no diagnostic value. This circumstance, most likely, is due to the use of a moderately sensitive method of determination, which has insufficient sensitivity for detecting explorative concentrations of troponin T.

In another study, Kubo et al. used a highly sensitive method for the determination of troponin T (hs-cTnT) in patients with HCM (n = 183). At the same time, increased levels of hs-cTnT were detected in 54% of patients [56]. Comparative analysis of several clinical studies demonstrates that the prevalence and degree of elevation in troponin T in patients with hereditary cardiomyopathies significantly differ depending on the use of moderately sensitive [55] and highly sensitive methods [56,57]. Thus, the capabilities of highly sensitive methods for detecting myocardial damage in hereditary cardiomyopathies are well above the capabilities of moderately sensitive methods. During the long mean follow-up period (4.1 ± 2.0 years), adverse cardiovascular events developed in 32% of patients with elevated hs-cTnT levels, and only in 7% of patients with normal serum hs-cTnT concentrations. According to multivariate analysis, elevated hs-cTnT levels represented independent predictors of adverse cardiovascular events (OR = 3.23) [56]. Thus, serum hs-cTnT concentrations have a high HCM predictive value. In another study, Frankenstein et al. determined hs-TnT in all patients (n = 24) with stable heart failure associated with dilated cardiomyopathy and noted the existence of short-term biological variations in hs-TnT levels [57].

Hładij et al. in their study found increased levels of hs-cTnT in 26 of 51 patients with HCM. Subsequently, the authors compared echocardiographic parameters used as risk factors for sudden cardiac death (SCD) among two groups of patients: (1) patients with elevated hs-cTnT levels (n = 26); and (2) patients with normal hs-cTnT levels (n = 25). Elevated hs-cTnT levels were found to be associated with all three echocardiographic parameters (risk factors for SCD) (maximum left ventricular thickness, provocable left ventricular outflow tract gradient (LVOTG), left atrial diameter) recommended by the European Society of Cardiology for assessing the prognosis of HCM patients [58].

Ho and colleagues studied the possibility of using laboratory biomarkers to identify individuals with overt HCM and those with asymptomatic HCM and sarcomere gene mutations preceding the development of HCM. Researchers measured cardiomarkers in patients with overt hypertrophic cardiomyopathy (n = 76), individuals with sarcomeric gene mutations (preclinical stage of hypertrophic cardiomyopathy) (n = 50), and a genotype-negative control group (n = 41) at rest and after challenge (physical activity). Patients with overt hypertrophic cardiomyopathy had significantly higher levels of N-terminal natriuretic peptide and hs-TnI both at rest and in response to exercise. However, there was no significant difference in the concentrations of these biomarkers between patients with preclinical HCM and control patients, both at rest and challenge tests with maximum exercise [59].

The mechanisms of elevation of cardiac troponins in HCM are not fully understood. The most likely is the input of the following mechanisms [52,54,57,58,59]: (1) apoptosis of cardiomyocytes; (2) imbalance between oxygen demand and delivery, caused by a sharp increase in the demand of hypertrophied myocytes against the background of possible compression of small branches of the coronary arteries, which leads to myocardial ischemia and damage to cardiomyocytes; and (3) with the progression of HCM, the terminal stage of heart failure develops, which leads to stagnation of blood in the vessels of the systemic circulation and damage to several organs, including the kidneys. Thus, in case of a complicated course, an additional mechanism for increasing troponins in the serum is activated, namely, a violated elimination of troponin molecules from the blood serum and their accumulation.

## 4. Causes and Mechanisms of Elevated Cardiac Troponins in Cardiac Arrhythmias

Elevated levels of cardiac troponins are often detected in cardiac arrhythmias (supraventricular tachycardia and atrial fibrillation), which should be considered in differential diagnostics. Zellweger et al. described four clinical cases of elevated troponin I with supraventricular tachyarrhythmia (heart rate (HR) = 170–250) with no coronary artery disease indications. In addition, three out of four patients showed an increase in the levels of total creatine kinase and creatine kinase-MB, which additionally indicates non-AMI-related damage to cardiomyocytes. The relationship between heart rate, tachycardia duration, and troponin I levels was not observed in this study [60].

In a larger study, Chow et al. analyzed the case histories of 78 patients with supraventricular tachycardia. The concentration of troponin I was above the 99th percentile (0.06 ng/mL) in 29 patients (37.2%), while the overall range of elevated values was 0.06–7.78 ng/mL. Elevated troponin I level in multivariate analysis were associated with an increased risk of adverse events (death, myocardial infarction, or hospital readmission for CVD-related causes) (OR = 3.67; 95% CI: 1.22–11.1; *p* = 0.02) [61]. Thus, this study showed a very significant prevalence of elevated cardiac troponin concentrations in patients with supraventricular tachycardia and revealed an association with an increased risk of future cardiovascular events.

Xue et al. described two clinical cases of increased cardiac troponins I in patients with paroxysmal supraventricular tachycardia. Both patients were admitted to the emergency room with chest pain complaints. The concentration of troponin I on admission was 0.09 ng/mL for the first and 0.16 ng/mL for the second patient (normal = 0.08). After a few hours, the troponin concentrations increased to 0.52 and 2.28, respectively, but the results of the electrocardiography and echocardiograms showed no significant abnormalities. However, an initially wrong ACS diagnosis was made. Within a week, both underwent coronary angiography, which showed absolutely intact coronary vessels. According to subsequent data, the patients were nevertheless given the correct diagnosis and passed radiofrequency ablation, after which both patients were safely discharged [62]. The findings of this study emphasize the need for careful interpretation of elevated cardiac troponin levels in pathologies not associated with ACS.

In a recent study by Gonzalez-Del-Hoyo et al., troponin I was elevated in 73 (32.9%) of 222 patients. Patients with high troponin levels were older. When assessing the long-term prognosis, five-year mortality was higher in the group of patients with elevated troponin (*p* < 0.001). Multivariate Cox regression analysis showed that troponin elevations were an independent predictor of all-cause death (OR = 1.95; 95% CI: 1.08–3.50, *p* = 0.026) in addition to age and prior heart failure [63].

Atrial fibrillation is one of the dangerous and common types of supraventricular tachyarrhythmias, peculiar by numerous chaotic and discoordinated contractions of the atrial fibers. In a large study, persistently elevated levels of cardiac troponins were observed in about half of patients with atrial fibrillation. Moreover, elevated troponin levels have been associated with an increased risk of stroke and mortality [64]. Nearly similar results have been obtained in several other studies [65,66].

The exact mechanisms of the release of cardiac troponins from cardiomyocytes in supraventricular tachycardia are undefined. As suggested by most authors, the reason for the release of troponins is associated with an imbalance between myocardial oxygen demand and the capability of oxygen delivery by the coronary arteries. As per the anatomical and physiological characteristics, the myocardium blood supply occurs in the relaxation phase (diastole). During systole, the opened three leaflets of the aortal valve occupy the three spaces of the Valsalva sinuses (aortal sinuses). In the area of two of them are the openings of the coronary vessels, which are thereby closed. In the diastolic phase, the aortal valve closes, and the lumen of the coronary arteries opens, into which blood rushes. Thus, conditioned by increased demand for oxygen and nutrients in tachycardia, due to the shortening of diastole, blood flow through the coronary arteries is reduced, which cannot fully meet the demand of the myocardium. Evidence for the involvement of this mechanism in increasing serum troponin levels is presented in a clinical study conducted by Ben Yedder et al. The researchers found increased troponin T levels in 24 of 73 subjects (32.9%) with supraventricular tachycardia. In this case, serious anomalies of the coronary arteries were excluded by coronary angiography and stress testing. It was found that in troponin-positive patients, the heart rate was significantly higher than in troponin-negative patients (190.8 versus 170.3; *p* = 0.008). Similarly, there was a significant correlation between the maximum heart rate during tachyarrhythmia and increased concentrations of troponin T (r = 0.637, *p* = 0.001) [67,68]. Further to these data, Ulimoen et al. reported that a decrease in heart rate also leads to a decrease in serum troponin T levels [69]. Thus, it can be assumed that a higher heart rate is associated with more pronounced myocardial ischemia and the degree of damage to cardiomyocytes. Nevertheless, in several studies [60,61,62,63], no correlation was found between the degree of increase in the marker and the duration and rate of tachycardia, which may indicate a certain role of supplementary mechanisms, in particular, the causes of tachycardia and individual morphological features; also, it should be kept in mind that the prevalence and degree of elevation of cardiac troponins concentration in supraventricular tachycardia significantly depend on the sensitivity of the determination methods [61,63,64,65,66,67]. Thus, highly sensitive methods for the determination allow more frequent detection of increased levels of troponins in patients with supraventricular tachycardia than when using moderately sensitive methods.

Thus, an increase in cardiac troponins in the blood serum in supraventricular tachycardia in some cases complicates the differential diagnosis of myocardial infarction and often leads to an erroneous diagnosis. On the other hand, the use of troponin tests has a high predictive value and can be used in clinical practice to manage patients with supraventricular tachycardia.

## 5. Diagnostic Value of Cardiac Troponins in Acute Aortic Dissection

There are few studies to measure the troponin levels in acute aortic dissection. The most comprehensive meta-analysis was conducted by Vrsalovic et al., reviewing four studies and 496 patients with acute aortic dissection. Elevated troponin was found in 26.8% and ranged from 23 to 33%. Elevated troponin levels were significantly associated with an increased risk of short-term death (OR 2.57; 95% CI 1.66–3.96): 40.6% of patients with acute aortic dissection and elevated troponin levels died, compared with 20.9% of negative troponin patients [70].

Li et al. first identified highly sensitive troponin T (hs-TnT) in patients with acute aortic dissection. Elevated hs-TnT levels (≥0.014 ng/mL) were observed in more than half of the patients (61.2%) [71]. Multivariate Cox regression analysis showed that hs-TnT is an independent factor for predicting intrahospital mortality (OR: 2.202, 95% CI: 1.111–4.367; *p* = 0.024). In the group of the deceased, hs-TnT concentrations were significantly higher than in the group of survivors (0.292 ± 0.516 versus 0.069 ± 0.154 ng/mL; *p* = 0.003). Hs-TnT values ≥ 0.042 ng/mL predicted short-term mortality in hospital with a sensitivity of 70.8% and a specificity of 76.4% [71].

Pourafkari et al. studied electrocardiographic and angiographic changes and the troponin T concentration in patients (n = 184: M = 120, F = 64) with acute aortic dissection. Ischemic ECG changes were registered in 38% of cases, while elevated cardiac troponin was in 36.6%. Nevertheless, the prevalence of elevated troponin T did not significantly differ in the groups of patients with and without ischemic changes on the ECG (*p* = 0.46) and in the groups with and without coronary artery lesions according to coronary angiography (*p* = 0.54), which indicates a non-coronarogenic (not associated with ischemia) increase in troponins [72].

The prevalence and degree of elevation in cardiac troponin levels depend on the sensitivity of the laboratory determination methods. For example, the highly sensitive method detects elevated levels of hs-TnT in more than 60% of patients with acute aortic dissection [71]. Whereas, when using moderately sensitive methods, elevated levels of cardiac troponins are found in about 30% of patients with acute aortic dissection [70]. Given that acute aortic dissection is an extremely life-threatening condition requiring immediate evaluation and therapy, biomarkers for stratification are required. The most valuable in this case are troponins, in particular, those determined by highly sensitive methods; however, due to the small number of such studies, further research is necessary. At the same time, additional problems in the differential diagnosis of myocardial infarction and acute aortic dissection arise with further consequences. For instance, thrombolysis, applied in the treatment of myocardial infarction, is contraindicated in dissecting an aortic aneurysm, since it increases hemorrhage in the aortal wall and contributes to its further rupture.

## 6. Causes and Mechanisms of Elevated Cardiac Troponins in Diseases of the Central Nervous System (Strokes, Subarachnoidal Hemorrhages)

Diseases of the central nervous system (strokes, subarachnoidal hemorrhages) have long been considered significant causes of increased cardiac troponins. According to a systematic review of 15 studies and 2901 patients with acute stroke over the period 2000–2007, the prevalence of elevated troponin values averaged 18.1% (95% CI 13.6–22.6). The percentage of results with elevated troponin was strongly influenced by the baseline characteristics of the subjects: studies that excluded patients with heart disease had a lower prevalence of positive troponin than the studies that did not exclude prior heart disease (10.2% (95% CI 6.0–14.5) versus 21.7% (95% CI 15.4–28.0)). Patients with acute stroke and elevated troponin were more likely to have signs of myocardial ischemia on an ECG (OR = 3.03; 95% CI 1.49–6.17) and had an increased risk of death [73].

**Figure 1:**
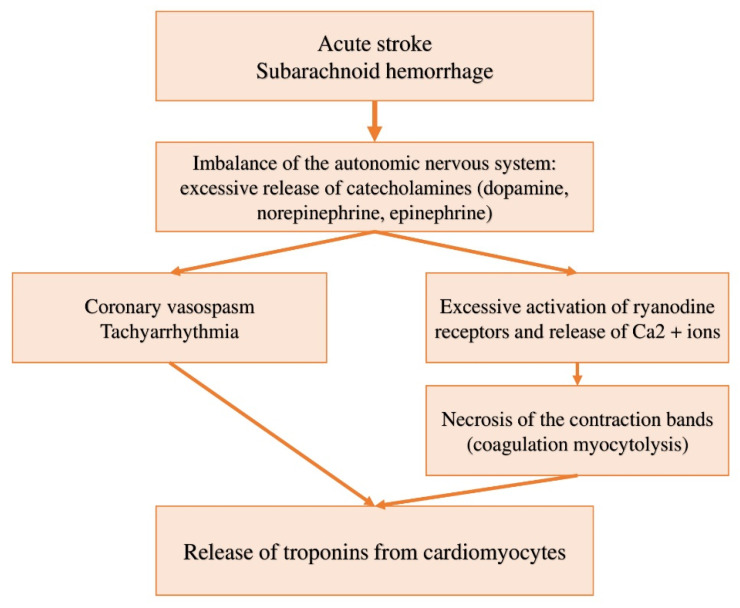
Pathophysiological mechanism of elevated cardiac troponins in stroke, subarachnoid hemorrhage, and stress cardiomyopathy. Modified and revised from [74,75].

The main causes and pathophysiological mechanisms conditioning the after-stroke increase in cardiac troponins are associated with impaired self-contained (autonomic) sympathetic and parasympathetic regulation of the myocardium. Excessive activation of the sympathoadrenal system is accompanied by an increased release of catecholamines (adrenaline, norepinephrine, dopamine) into the blood and the synaptic gap. Excessive concentration of catecholamines disrupts the course of metabolic reactions of the myocardium and also causes vasoconstriction, which also indicates the contribution of the ischemic mechanism to troponin elevation (Figure 1). Catecholamines, secreted at the synapses of the intracardiac nerves, have an activating effect on calcium channels, which leads to excess calcium intake, metabolic disorders, and muscle relaxation. In the histopathological picture of the myocardium during strokes, there are areas of myofibrillar degeneration (also known as coagulation myocytolysis or contractile zone necrosis). In this case, cardiomyocytes die in a state of hypercontraction with noticeable contraction bands, which occurs within a few minutes and is associated with early calcification and mononuclear infiltration. This feature distinguishes this type of cell death, from necrosis of cardiomyocytes in ischemia, when cells die in a relaxed state without noticeable contractions, and late-onset of calcification, which is referred to as coagulative necrosis. In animal models using stress hormones and infusion of catecholamines, myofibrillar degeneration has been replicated, and it has been noted that areas of necrosis occur near the intracardiac nerves [74,75]. In the case of subarachnoidal hemorrhages, blood enters the spaces (ventricles) of the brain, increasing intracranial pressure and mechanically affecting the centers of the autonomic nervous system (hypothalamus and brain stem), which leads to excessive release of catecholamines [74].

According to a retrospective study by Cui et al., elevated troponin I levels were observed in 18.5% of patients with acute ischemic stroke. Patients with elevated troponins were more likely to have hypertension and were older, had elevated levels of D-dimer, inflammatory cytokines, and decreased renal function (decreased glomerular filtration rate), which is one of the pathomechanisms leading to increased troponins. In addition, troponin-positive patients had a poor short-term prognosis [76].

Ahn et al. evaluated the long-term survival of troponin-positive patients with ischemic stroke, which the authors themselves report was the first time. An increase in troponins in patients with an acute stage of ischemic stroke (n = 1692) is associated with long-term death: up to 1 year after, patients more often died from stroke and oncology; after 1 year, death from heart failure prevailed over a 6-year period [77].

Atrial fibrillation is one of the leading causes of death from ischemic stroke. Beaulieu-Boire et al. reported that elevated troponin I in patients with strokes and transient ischemic attacks can predict the occurrence of atrial fibrillation detected by 24 hours Holter monitoring. In addition to the predictive role of elevated troponin I in atrial fibrillation, the authors noted its association with a higher risk of 3-month death compared to troponin-negative patients (50.0% versus 16.1%; *p* = 0.0001) [78].

Persistently elevated concentrations of highly sensitive troponins (>99th percentile) indicate chronic myocardial damage. Ryden et al. found a relationship between elevated hs-cTnT concentrations and the risk of stroke in 19,460 patients, among which 1528 (7.9%) had chronic myocardial damage. During a mean follow-up period of 2.1 years, stroke occurred in 244 patients (1.2%). At the same time, the risk of stroke increased 4 times in patients with an hs-cTnT concentration > the 99th percentile, compared with patients whose hs-TnT level remained <5 ng/L [79].

Vafaie et al. evaluated the relationship of elevated hs-TnT concentrations in patients with atrial fibrillation to the possibility of stroke. The study group included 2898 patients who were admitted to the emergency cardiology department of the University Hospital Heidelberg (Germany) with atrial fibrillation. Multivariate Cox regression established an association of increased hs-TnT concentrations > the 99 the percentile (14 ng/mL) with the risk of stroke (OR = 2.35 95% CI: 1.26–4.36, *p* = 0.007). The authors believe measuring hs-TnT may improve the prediction of stroke risk in patients admitted with atrial fibrillation compared to traditional risk stratification on the CHA2DS2-VASc scale based on clinical variables alone [80].

Thus, the measurement of cardiac troponins can be useful for detecting heart problems associated with stroke, as well as predicting both short-term and long-term mortality after a stroke, which, accordingly, should be considered in treatment. Highly sensitive troponins are valuable in stratifying the risk of stroke; however, further study and clarification are needed.

## 7. Causes and Mechanisms of Elevated Cardiac Troponins While Using Medications with Cardiotoxic Properties

Against the background of improving the diagnostic and treatment process in oncology, a significant increase in the risk of various adverse effects, in particular, those related to the cardiovascular system, namely, cardiac dysfunction, arterial hypertension, vasospastic and thromboembolic ischemia, arrhythmias, etc., has been observed [81,82,83,84]. Many medications have cardiotoxic properties, but chemotherapeutic agents, especially the group of anthracyclines, are most aggressive towards the myocardium. Developing undesirable effects can lead to disability and death of patients after cancer treatment. The importance of the problem of cardiotoxicity is explained by the emergence of an interdisciplinary department—cardioncology—which entails the creation of specialized cardio-oncology departments [85,86] and the development of separate recommendations for the management of such patients [87].

Suter et al. divide all chemotherapy medications into two groups according to their damaging effect on the cardiovascular system: (1) medications that cause the death of cardiomyocytes and irreversible dysfunction of the myocardium; and (2) medications that cause temporary (reversible) dysfunction without further consequences after the completion of the course [88]. The mechanisms of increasing the concentration of cardiac troponins in the blood serum may be associated with oxidative stress, apoptosis processes, ischemia due to an imbalance between oxygen demand and supply, as well as the direct cytotoxic effects of medications [81,82,83,84].

Guidelines from the European Society for Medical Oncology (ESMO) recommend the use of troponin assays for patients undergoing chemotherapy, especially anthracyclines [89].

Several studies have demonstrated the high diagnostic value of highly sensitive troponins and their advantages over the use of moderately sensitive methods for the determination of cardiac troponins [90,91,92,93]. For example, Sarzhevsky et al. determined the troponin T, troponin I (high-sensitive method), and N-terminal brain natriuretic peptide (NT-proBNP) in patients with malignant lymphoproliferative diseases (Hodgkin’s lymphoma and non-Hodgkin’s lymphomas) during high-dose chemotherapy and autologous hematopoietic stem cell transplantation. On admission to the hospital, the concentrations of cardiac troponins were normal in all patients. An increase in troponin T was recorded in only 2 of 56 patients (3.6%) and increased hs-TnI levels in 27 of 101 patients (26.7%). A noteworthy observation regarding the gender characteristics of hs-TnI was made, namely that, in women, it was increased in 34.3% of cases, and in men by 11.7% (*p* = 0.015). On Day 12 after anthracycline treatment, the troponin T levels completely normalized, while hs-TnI remained elevated in 11 patients (10.9%) [90]. This study demonstrates the significant superiority of highly sensitive troponin measurement methods for detecting cardiotoxicity compared to moderately sensitive methods.

In another work, Jones and colleagues compared the hs-cTnI concentrations in patients receiving anthracyclines (doxorubicin) (n = 38) with the control group (n = 46). The authors also examined the correlation between medication dose and the degree of increase in serum hs-cTnI levels. At the same time, the hs-cTnI concentrations in the anthracycline cohort were significantly higher than in the non-anthracycline cohort (all values were below the 99th percentile). The correlation between doxorubicin dose and hs-TnI concentration was very low (r < −0.22) [92].

Hole described a case of cardiotoxic effect in a 41-year-old man using methylphenidate (Ritalin), while the clinical picture (tachycardia, chest pain) and troponin I concentration (0 h–82 ng/L, 6 h–616 ng/L, and 12 h–529 ng/L, with the norm = 30 ng/L) in dynamics corresponded to the diagnosis of myocardial infarction. The acute coronary syndrome was ruled out by coronary angiography. Considering the mechanism of action of this medication, the authors suggested the temporary occurrence of vasoconstriction of the coronary arteries against the background of tachycardia of increased myocardial oxygen demand [94].

In a recent study, it was also found that the use of statins is accompanied by an increase in the concentration of hs-cTnI in the blood serum [95], which may indicate the presence of potential cardiotoxic effects [96].

Thus, laboratory determinations of the concentrations of cardiac troponin molecules, performed by the highly sensitive methods, are capable of registering even minor (asymptomatic) myocardial damage. At the moment, the availability of pronounced cardiotoxic effects has been proven in many chemotherapeutic medications used to treat cancer. At the same time, the presence of cardiotoxic properties in some other medications applied is very likely, which makes their study essential to adjust for safe dosages. This is especially true for groups of patients with a reduced reserve capacity of the cardiovascular system and additional factors in the development of myocardial infarction.

## 8. False-Positive Causes of Elevated Cardiac Troponins

In addition to the above reasons for the increase in cardiac troponins due to the direct or indirect effects of factors damaging the myocardium, it should be kept in mind that the increase in the levels of cardiac troponins may be due to false-positive causes and mechanisms. In particular, some influencing pre-analytical (pre-laboratory) and analytical factors can falsely overestimate the results in the absence of asymptomatic myocardial damage. Combinations of several minor interfering factors, especially those that arise against the background of asymptomatic myocardial damage, can also lead to an excess of the diagnostic threshold and a misdiagnosis. The possibility of obtaining a false-positive result is reflected in the term “99-percentile”, according to which 1 person out of 100 (1%) will have an unreasonably overestimated result [97,98]. In real clinical practice, false-positive results can be much more common.

The most common causes of falsely high troponin concentrations at pre-laboratory and laboratory stages are heterophilic antibodies and autoantibodies, in particular, rheumatoid factor, immune complexes, cross-reactions with skeletal muscle troponins, fibrin clots, microparticles, endogenous interference (alkaline phosphatase, hemolysis, and others), as well as malfunction of the analyzer (mechanical failures and calibration errors, use of expired reagents, etc.) [99,100,101,102,103,104,105,106].

### 8.1. Heterophilic Antibodies 

Heterophilic antibodies (Greek getero—different, phile—affinity) include two types: (1) “true” heterophilic antibodies are formed in response to poorly recognized antigens, as a rule, to foreign animal proteins, possess a weak avidity, but multi-specific (polyvalent) activity; and (2) human anti-mouse antibodies are generated against well-defined antigens (monovalent), for example, against monoclonal mouse sera, and are characterized by strong avidity. Both, under certain conditions, can become a source of significant interference in immunochemical methods for the analysis of many parameters, including cardiac troponins T and I.

Heterophilic antibodies are produced by B-lymphocytes of the human immune system in response to exposure to various antigens, such as blood transfusion, vaccines (vaccinations), contact with animal antigens (pets, mice, rabbits, etc.), persistence of viral infections, therapeutic use of mouse monoclonal antibodies, or incompletely humanized (human) antibodies, etc. [99,100,101,102].

Lippi et al. described a clinical episode of false elevation in cardiac troponin I and conducted a systematic literature review for the period 1998–2012. During this time, the researchers found 16 original studies and clinical cases that noted the false-positive effect of heterophilic antibodies on the concentration of cardiac troponins. According to the literature data obtained, the frequency of false-positive results is 0.1–3.1% in the general population; however, it can be significantly increased, up to 50% in a specific category of patients. Heterophilic antibodies can affect both the troponin I and T of any test system. This type of interference is practically unpredictable; for example, it cannot be detected by visual inspection of the sample, unlike hemolysis or fibrin clots. A false-positive result due to heterophilic antibodies can only be suspected by clinicians since it is they who receive information about all diagnostic methods for a given patient. However, even in the case of a clear discrepancy between the result of troponin T or I, clinical and instrumental data, and/or the results of other cardiomarkers (creatine kinase-MB, another type/method for determining troponin, etc.), the final confirmation requires time and expansion of extra resources [99]. A clear false-positive result is reported to the laboratory, where serial dilutions of the sample can be made. If non-linear (non-correlated) results are obtained, a false-positive result is assumed due to heterophilic antibodies. For the final confirmation of such a false-positive result, it is necessary to use reagents that block heterophilic antibodies. Decreasing the result with the addition of such an antibody blocker indicates the presence of interference of heterophilic antibodies.

A study by Zaidi et al. reported a false-positive increase in troponin I in a 53-year-old woman. The patient was admitted three times during the year with chest pains; however, there were no ischemic changes on the ECG, and the angiography showed intact coronary vessels. However, the concentration of troponin I on admission was 0.37, which is 5 times higher than the reference (0–0.069). A blood sample from a patient sent to another laboratory to test for a different type of troponin (troponin T) showed normal results. Further investigation and treatment of this sample with heterophilic antibody blockers confirmed the presence of a false–positive increase in troponin I [100]. In a study by Lahat et al., events developed in approximately the same scenario. A 58-year-old man was repeatedly hospitalized due to elevated troponin I, despite the absence of ischemic changes on the ECG and coronary angiography. Using another commercial kit for troponin I, as well as measuring troponin T, the results expectedly turned out to be negative, based on which it was concluded about the interfering effect of heterophilic antibodies [101].

In general, strategies for dealing with heterophilic antibodies include (1) testing the sample on a different analyzer and/or using another cardiomarker (for example, creatine kinase-MB) if the results are equivocal; (2) serial dilution of the sample and assessing the linearity of the results; and (3) adding heterophilic blocking reagents or pooled animal sera into a sample [99,100,101,102,106,107,108]. Nguyen et al. believe that the prevalence of false-positive results, both troponins and other indicators, will increase in the future due to the emergence of immunotherapy in the treatment of a wide range of conditions and the use of radioactively labeled antibodies in diagnostic immunoscintigraphic procedures [109].

### 8.2. Autoantibodies

Rheumatoid factor (RF) means autoantibodies of the IgM class directed against their own antibodies (immunoglobulins) of the IgG class. Produced in large quantities in autoimmune diseases (rheumatoid arthritis, systemic lupus erythematosus, polymyositis, etc.), rheumatoid factor usually binds to IgG Fc fragments, forming immune complexes that, in addition to damaging the body, have an interfering effect on laboratory results, including the number of troponin tests.

A multicenter study of the influence of the rheumatoid factor included 10 donors. Their blood serum samples were sent to 66 clinical laboratories, in which a total of 74 indicators (hormones, vitamins, tumor markers, cardiomarkers, etc.) were examined. Of the 3445 results, approximately 8.7% were considered false positives, with many resulting in clinically significant overestimations. About half of the false positives would potentially be misleading in diagnosis and would not be corrected by the addition of a heterophilic antibody blocking reagent [110].

Another study examined 12 serum samples with positive rheumatoid factor and no evidence of myocardial infarction. Serum samples from 7 of 12 patients containing rheumatoid factors had measurable troponin I concentrations, while 4 patients had a troponin I level above 2.0 ng/mL. At the same time, troponin T, measured by another method, did not reveal a single case of a false increase. After the addition of a polyclonal anti-rheumatoid factor antiserum, false-positive troponin I results were eliminated. Therefore, the results of elevated troponin concentrations in patients with elevated rheumatoid factor levels should be interpreted with caution [111]. However, the rheumatoid factor does not always lead to false-positive results even at very high concentrations. Thus, Kenny et al. found no elevated troponin I level in patients with rheumatoid arthritis (n = 60, rheumatoid factor concentration range = 15–2724 IU/mL) when using two commercial kits (Abbott AxSYM, Bayer ADVIA Centaur) [112].

Al-Awadhi (2007) measured troponin I with the Beckman Access test system in patients with autoimmune diseases: seropositive rheumatoid arthritis (RA) (n = 50), seronegative RA (n = 50), systemic lupus erythematosus (n = 50), primary Sjogren’s syndrome (n = 20), and Graves’ disease (n = 15). Of the 50 patients with seropositive rheumatoid arthritis, 5 subjects had troponin I concentrations higher than the diagnostic value for myocardial infarction. At the same time, in no patient with seronegative RA and other autoimmune diseases, troponin I values went beyond the reference limits. The authors noted that in univariate regression analysis, troponin I and rheumatoid factor concentrations in patients with seropositive RA were positively associated (r = 0.35; *p* < 0.02) [113].

Thus, the rheumatoid factors can have different effects on the troponin results, and the frequency of such cases is also highly variable depending on the equipment. It is also notable that several studies have had small patient populations, which may also play a role. Larger population studies are needed for each specific test system used to confidently say that there is no interference.

Cross-reactions of the diagnostic antibodies of troponin immunoassays with skeletal muscle troponin isoforms arising in diseases and injuries of skeletal muscles (myopathy, rhabdomyolysis, etc.) were considered frequent when using “outdated” immunoassays of the first and second generation [114,115,116,117,118,119]. Nevertheless, several cases have been described in which modern test systems, including monoclonal antibodies that are part of modern highly sensitive methods, also reacted to skeletal troponin isoforms, being more typical for troponin T [120,121]. At the same time, there is evidence for the presence of the expression of cardiac troponin isoforms in skeletal muscle, which, however, requires further studies to be confirmed.

### 8.3. Alkaline Phosphatase

The concentration of alkaline phosphatase can significantly increase in several pathologies (hepatobiliary diseases, such as obstructive jaundice, biliary cirrhosis of the hepatic, intrahepatic cholestasis, viral hepatitis, and liver cirrhosis; bone diseases; hyperparathyroidism). Alkaline phosphatase is commonly used to enhance the signal in some immunoassays. Some studies have demonstrated the effect of endogenous alkaline phosphatase on the results of cardiomarkers, including troponins. Butch et al. were among the first to describe the effect of alkaline phosphatase on the concentration of creatine kinase-MB-mass, determined on the Stratus immunochemical analyzer [122]. Dasgupta et al. observed a very significant effect of alkaline phosphatase on the concentrations of troponin I (Dade Stratus). Thus, the initial serum sample had a troponin I concentration of 0.5 ng/mL with an alkaline phosphatase activity equal to 46 U/L. By adding different amounts of a standard solution of alkaline phosphatase to this sample, the researchers recorded significant changes in troponin I levels. At an alkaline phosphatase activity of 129 U/L, the troponin I level falsely rose to 4.3 ng/mL. With a further increase in alkaline phosphatase activity to values of 222 U/L, 445 U/L, and 913 U/L, the troponin concentrations increased to 9.4 ng/mL, 20.4 ng/mL, and 40.1 ng/mL, respectively. When using a test system (Bayer Diagnostics, Tarrytown, NY, USA) that did not contain alkaline phosphatase, the addition of this enzyme to the studied serum did not change the troponin I concentrations [123].

Marinheiro et al. reported falsely elevated troponin I in an 18-year-old female patient who was hospitalized several times for three years for recurrent myopericarditis. The troponin I concentrations on admission were always positive (maximum value 3.1 ng/mL). During the next hospitalization due to complaints of palpitations and mild chest pain, the troponin I value on admission was 1.31 ng/mL; however, on the following days of hospitalization, the troponin I levels were negative. Physical examination, ECG, echocardiography, and cardiac MRI showed no signs of active myocarditis. Based on this, the doctors had assumptions about the falsely high result. To exclude a false-positive cause of an increase in troponin I, two blood samples were taken from the patient simultaneously and sent to two different laboratories: (1) a laboratory using the Beckman Coulter Access AccuTnI + 3 test system (Marseille, France); and (2) a laboratory using the Abbott Architect STAT hs-TnI immunoassay (Wiesbaden, Germany). In the first laboratory, the troponin I result was significantly higher than normal (1.03 ng/mL), while in the second laboratory, the troponin levels were negative (hs-TnI = 0.00 ng/mL). Even though the activity of alkaline phosphatase was within the normal range (58 U/L, N = 40–150 U/L), it was proved in the central laboratory of Beckman Coulter that falsely elevated troponin I is associated with the influence of this enzyme [124].

Herman et al. also reported that alkaline phosphatase-based immunoassays lead to falsely elevated levels of not only troponin I but also other indicators, in particular, human chorionic gonadotropin. The authors recommend using alternative methods for the determination of troponins for those patients in whom the alkaline phosphatase activity and troponin concentration are significantly increased. Ultimately, it is recommended to switch to methods that do not use reagents with endogenous homologous compounds, as they are less prone to false results [125].

Given the widespread use of quantitative troponin tests, false-positive results can harm the patient and/or lead to unnecessary costs (unnecessary non-invasive methods, invasive interventions, hospitalization) [126,127,128]. However, due to a large number of manufacturers and the variety of immunological tests and devices, the true frequency of false-positive results is unknown, and it is almost impossible to identify all the interfering factors, due to the economic component of large-scale population studies in each specific laboratory.

## 9. Conclusions

The reasons and mechanisms of pathological increase in cardiac troponins considered in this article (diabetes mellitus, arterial hypertension, hereditary cardiomyopathies, cardiac arrhythmias, acute aortic dissection, diseases of the central nervous system), not associated with acute myocardial infarction, are of great practical importance. They should be considered by practicing physicians when making a differential diagnosis between acute myocardial infarction and other pathological conditions. Elevated levels of cardiac troponins, in particular highly sensitive cardiac troponins, in the pathologies discussed in this article have prognostic value and can be used in practical medicine. Hence, elevated levels of cardiac troponins in cardiac and non-cardiac diseases reported in this article (diabetes mellitus, arterial hypertension, hereditary cardiomyopathies, cardiac arrhythmias (atrial fibrillation, supraventricular tachycardia), acute aortic dissection, diseases of the central nervous system (strokes, subarachnoid hemorrhage)) can be used to identify patients with a poor prognosis, providing additional help to clinicians in choosing or modifying patient management tactics. Besides, the opportunity to study the urine cardiac troponins’ pathological conditions that cause damage to cardiomyocytes is a very interesting direction for future research. Obtaining this biomaterial has several advantages over blood serum (non-invasiveness, painlessness, reduced risk of infection with blood-borne infections, as well as the ease of obtaining such a biomaterial) and the potential to replenish an array of existing diagnostic methods. The causes and mechanisms of false-positive increases in troponin levels deserve special attention from physicians and researchers.

## Data Availability

Not applicable.
